# Acupuncture of the adductor magnus for the treatment of post-stroke equinovarus: an investigation of the mechanism of action based on the anterior deep line

**DOI:** 10.3389/fneur.2025.1696812

**Published:** 2025-11-05

**Authors:** Bingxin Yu, Xiuying Teng, Lina Lu

**Affiliations:** ^1^The Second Clinical College, Heilongjiang University of Chinese Medicine, Harbin, Heilongjiang Province, China; ^2^The Second Department of Rehabilitation Medicine, The Second Affiliated Hospital, Heilongjiang University of Chinese Medicine, Harbin, Heilongjiang Province, China

**Keywords:** post-stroke equinovarus, acupuncture, adductor magnus, myofascial chain, Deep Front Line, neuroplasticity

## Abstract

Post-stroke equinovarus foot is a prevalent motor dysfunction among stroke survivors. Its pathophysiology involves an imbalance of muscle tone, spasticity, and a subsequent disruption of the biomechanical chain resulting from central nerve injury. Conventional rehabilitation methodologies predominantly emphasize the restoration of localized muscular function, while interventions targeting motor pattern abnormalities resulting from systemic fascial conduction remain underdeveloped. In recent years, the fascial chain theory has provided significant anatomical and biomechanical perspectives for understanding the integrity and continuity of human movement. This paper proposes a novel integrative mechanistic framework, based on the fascial chain theory, in which needling the adductor magnus regulates the Deep Front Line (DFL) to improve the equinovarus foot posture. We hypothesize that the effects of acupuncture on the DFL of the vastus lateralis muscle are mediated through biomechanical and neuroplastic pathways: a mechanical pathway of fascial tension redistribution, a neuroplastic pathway of proprioceptive recalibration, and a resultant biomechanical pathway of gait optimization. While fascial chains are well-described anatomically, their therapeutic exploitation via acupuncture remains unexplored in the context of stroke rehabilitation. A thorough review of extant literature was conducted to demonstrate the scientific validity of this hypothesis across these three levels. This paper presents a novel perspective on the rehabilitation of equinovarus foot following a stroke and provides a foundation for future validation studies.

## 1 Introduction

Stroke, a neurological disease that significantly impacts quality of life, often leads to equinovarus foot as a common clinical sequela. Statistics indicate that the incidence of post-stroke equinovarus ranges from 18 to 56% (1). This condition not only severely affects the patient’s walking ability and gait stability but may also cause secondary complications such as pain and falls (2). Central nervous system injury following stroke results in upper motor neuron syndrome, characterized by impaired corticospinal tract conduction, reduced descending inhibitory pathways, and hyperactive spinal stretch reflexes (3). This neuromotor imbalance significantly lowers the stretch reflex threshold of the posteromedial calf muscles—including the tibialis posterior, soleus, and flexor hallucis longus—leading to persistent spasticity and abnormal synergistic activation. Concurrently, the lateral muscle group, particularly the peroneus longus and brevis, experiences weakened muscle strength due to denervation or disuse atrophy, failing to effectively counteract the overactivity of the invertor muscles (4, 5). Traditional rehabilitation interventions—such as strength training, stretching, orthoses, and neurodevelopmental techniques like Bobath and Proprioceptive Neuromuscular Facilitation (PNF)—have demonstrated certain efficacy (6, 7). Meanwhile, interventions in alternative medicine have also demonstrated comparable effects in both neurological and biomechanical aspects (8). However, these approaches often focus on isolated muscles or local joint function and remain limited in their systematic and sustained management of movement disorders resulting from whole-body abnormal tension transmitted through fascial networks.

In recent years, the concept of fascial chains, informed by Ida Rolf’s structural integration principles and Thomas Myers’ systematic elaboration on myofascial continuity, has provided a novel perspective for understanding the integrity of human movement and the mechanisms of abnormal force transmission (9). This theory posits that the human body is not merely an assembly of isolated muscles but rather a series of interconnected functional units linked by a fascial network, spanning joints and muscle groups to form myofascial chains (10). Among these, the Deep Front Line (DFL), as the deepest longitudinal mechanical pathway traversing the torso and limbs—extending from the medial foot to the cranial base—plays a crucial role in maintaining core stability and longitudinal tension balance of the body (9). Acupuncture, a key component of traditional Chinese medicine, employs its unique meridian theory and stimulation of surface points not only to regulate qi and blood flow and balance yin and yang but also to modulate mechanical and physiological functions of the entire fascial network (11, 12).

Targeting the adductor magnus—a key node within the DFL—as a site for acupuncture intervention offers a promising and systematic strategy for correcting post-stroke equinovarus. This paper aims to elucidate the central role of the adductor magnus within the DFL and explore the theoretical basis and multidimensional mechanisms of acupuncture applied to this muscle, thereby providing comprehensive and in-depth theoretical guidance for clinical practice.

## 2 The anatomical foundations of the myofascial chain theory and the DFL

### 2.1 Core concepts and development of fascial chain theory

The emergence of fascial chain theory represents a significant breakthrough beyond traditional isolated muscle anatomy and exercise physiology. Its origins can be traced back to Franz Reuleaux’s mechanical concept of “linkage,” which emphasized the interconnectedness and interdependence of structures, later widely adopted and applied in other fields (13). Further contributions came from Dr. Vladimir Janda’s explanation of the “chain reaction” doctrine, which posits that motor dysfunctions often follow a domino effect, meaning that a problem in one segment can trigger a series of reactions (14). However, it was Dr. Ida Rolf who truly systematically proposed and developed the concept of fascial chains through her “Structural Integration” approach. She observed the body as a whole, with muscles and fascia interconnected, forming continuous pathways, and emphasized that manual adjustments could correct bodily imbalances (15).

In his book Anatomy Trains, Thomas Myers further systematized and popularized the fascial chain theory through extensive anatomical practice. He divided the human myofascial system into multiple “myofascial meridians” that traverse the body, identifying key pathways such as the “Superficial Back Line, Superficial Front Line, Lateral Line, Spiral Line, Arm Lines, Functional Lines, and Deep Front Line” (15). Myers emphasized that these myofascial chains are not isolated anatomical structures but rather a three-dimensional network formed by continuous fascial tissues enveloping and connecting bones, muscles, nerves, and blood vessels. This network playsa central role in maintaining posture, transmitting mechanical loads, absorbing impact, and enabling fine motor control. The core idea is that the continuity of the myofascia is fundamental to coordinated human movement and balance, and abnormal tension in any segment can affect the entire chain through fascial transmission. With further research, multiple scholars (16, 17) have explored the biomechanical properties of fascia, such as elastic modulus, viscoelasticity, and neural innervation, revealing fascia’s role as an active organ in biomechanical signal transmission and proprioception, thereby enriching the theoretical foundation of fascial chains. The coordinated function of these chain structures in the sagittal, coronal, and horizontal planes underpins stable and coordinated human movement.

### 2.2 Anatomical structure and function of the Deep Front Line

The DFL is the most central and profound longitudinal myofascial pathway in fascial chain theory, regarded as the “core” structure of the body. It originates from the plantar fascia on the medial side of the foot, including the tendons of the tibialis posterior, flexor hallucis longus, and flexor digitorum longus. It then ascends through the deep muscles of the medial calf, such as the tibialis posterior and flexor digitorum longus, continues upward through the medial femoral muscle group and the adductor magnus, passes via the pelvic floor muscles and the anterior longitudinal ligament of the spine, proceeds through the iliopsoas and the diaphragm, connects cranially to the deep fascia of the thoracic cavity and neck, and finally terminates at the hyoid muscle group and deep cervical structures at the base of the skull (9). The DFL plays a critical role in maintaining core stability, postural control, and mechanical force transmission between the lower limbs and the trunk. Structures within the DFL, such as the diaphragm, pelvic floor muscles, and deep spinal muscles, collectively form a “core stability system” that maintains intra-abdominal pressure, stabilizes the spine, and provides a solid supportive foundation for the body. Furthermore, as an efficient mechanical transmission pathway, the DFL effectively conveys forces from the lower limbs to the trunk and reciprocally transmits motor commands from the trunk to the lower limbs (18). Simultaneously, tension balance within the DFL contributes to the maintenance of sagittal plane equilibrium. Abnormal tension or structural alterations in any segment may propagate proximally or distally through the continuous network of the DFL, thereby affecting overall mechanical balance and posture, and potentially leading to compensatory movement patterns. Following stroke, the anatomical continuity of the DFL serves as the pathological basis for abnormal tension transmission. Upper motor neuron lesions lead to an imbalance in the gamma motor system, triggering spasticity of the adductor magnus muscle. This resultant hypertonia may significantly increase collagen cross-linking within the adductor magnus tendon and its surrounding fascia, thereby reducing the gliding capacity of the DFL (19). This aberrant tension is subsequently transmitted along the iliopsoas-tibialis posterior myofascial continuity to the deep posterior compartment of the leg, further exacerbating spasticity in the tibialis posterior muscle. This cascade ultimately initiates or aggravates an equinovarus foot deformity.

### 2.3 The central role of the adductor magnus in the pathogenesis of post-stroke equinovarus foot

The adductor magnus, as the largest adductor muscle in the human body, features tight fascial integrations with the deep fascial structures of the medial lower limb. Functionally, it acts synergistically to maintain the mechanical equilibrium of the lower extremity (20, 21). Following a stroke, upper motor neuron injury frequently induces spasticity and abnormal hypertonicity in the adductor magnus. This can subsequently lead to adaptive shortening of muscle fibers, collagen deposition, and fascial adhesions, which significantly increase the elastic modulus of the local fascia and alter its gliding properties. Histologically, these alterations may be accompanied by changes in the rheological properties of the extracellular matrix, resulting in the formation of palpable tense fascial bands or nodules (22). According to the theory of myofascial chains, this aberrant tension is transmitted proximally and distally along the DFL. Regarding proximal transmission, the contracted adductor magnus may, via its connections to the pelvis and the anterior longitudinal ligament of the spine, contribute to an increased anterior pelvic tilt. This alters the mechanical position of the hip joint, adversely affecting lower limb alignment and gait (23). Simultaneously, pertaining to distal transmission, the abnormal tension may propagate along the iliopsoas-tibialis posterior myofascial continuity to the medial aspect of the leg, thereby indirectly influencing the tibialis posterior muscle (9) and leading to a significant increase in its tension. Hyperactivity of the tibialis posterior generates a powerful plantar flexion-inversion moment at the ankle joint, while concurrently inhibiting the synergistic contraction of its antagonists, the peroneus longus and brevis muscles. This establishes a biomechanical pattern characterized by “medial spastic dominance and lateral inhibitory weakness,” resulting in a medially shifted force line (24). The ultimate consequences are limited ankle dorsiflexion and a pronounced equinovarus foot deformity. This condition manifests clinically as increased weight-bearing on the medial foot, difficulty in shifting the center of gravity forward, reduced cadence, and toe drag—cardinal features of post-stroke equinovarus gait. The pathological changes in the adductor magnus may thus represent a critical node for tension imbalance within the DFL. Through its interactions with the fascial network, it potentially transmits abnormal tension and participates in adaptive changes within central neural function. Collectively, these mechanisms are posited to contribute to and exacerbate the development and progression of post-stroke equinovarus foot.

## 3 Mechanism of action

### 3.1 Mechanical restructuring effects

The mechanical stress generated by needle insertion and manipulation during acupuncture can be regarded as a localized trigger for mechanical restructuring. This mechanical stimulus can locally upregulate matrix metalloproteinase-3, facilitating the degradation of excessively deposited collagen (25). We hypothesize that such extracellular matrix (ECM) remodeling may not only contribute to a reduction in the macroscopic mechanical stiffness of the DFL but is also anticipated to improve the rheological properties of hyaluronic acid within the ECM (26), although direct empirical evidence measuring fascial tension changes across the entire DFL following acupuncture remains to be established. Collectively, these microscopic changes are believed to reduce sliding resistance between fascial layers, facilitating smoother gliding of muscle fibers, thereby aiding in the alleviation of abnormal mechanical constraints and contractures within the DFL. Following the improvement in fascial elasticity and gliding capacity, the mechanical restructuring effects are thought to propagate to mesoscopic and macroscopic levels (27, 28). After a stroke, hyperactivation of the adductor magnus transmits abnormal tension distally along the myofascial chain, disrupting the mechanical balance of peri-ankle muscle groups. Acupuncture targeting the adductor magnus may release its excessive tension, mitigate this abnormal distal pull, and thus create favorable conditions for restoring the normal tension balance between the adductor muscle group and its antagonists—the tibialis anterior and peroneus longus and brevis muscles. The reestablishment of this local intermuscular mechanical balance forms the foundation for optimizing the synergistic function of the entire lower limb kinetic chain, thereby supporting critical movements such as ankle dorsiflexion and eversion during the gait cycle. Simultaneously, hypertonicity and contracture of the adductor magnus often lead to aberrant pelvic biomechanics (18). By alleviating its excessive pull on the pelvis, acupuncture may assist in correcting anterior pelvic tilt and restoring a more normative pelvic posture and weight distribution. This optimization of pelvic alignment provides a more stable and efficient mechanical foundation for lower limb movement, indirectly enhancing the coordination of ankle motion.

### 3.2 Neuroplasticity modulation

Stroke-induced attenuation or distortion of proprioceptive input significantly impairs the central nervous system’s ability to perceive and regulate muscle and joint states (29). Under pathological conditions, abnormal mechanical forces may activate mechanosensitive Piezo2 ion channels in the dorsal horn of the spinal cord (30, 31), contributing to central sensitization and inducing long-term potentiation (LTP) in C-fibers (32). These mechanisms may concurrently disrupt 5-HT/GABAergic descending inhibitory pathways (33), collectively leading to a reduced excitation threshold of γ-motor neurons and increased firing frequency of muscle spindles. This cascade promotes the formation of a pathological “spasticity-contracture” movement pattern. Acupuncture intervention can enhance and provide precise sensory input by activating deep proprioceptors within the adductor magnus and cutaneous/tactile receptors in the skin and fascia (29). According to the principles of neuroplasticity, such input effectively promotes functional reorganization of the nervous system (34). These signals can be received and integrated more efficiently by the spinal cord and cerebral cortex (35), thereby improving the perception and control accuracy of lower limb movements. Simultaneously, acupuncture may modulate the expression of brain-derived neurotrophic factor (BDNF) and its TrkB signaling pathway (36), reversing neurotrophic dysfunction caused by abnormal stress and mediating synaptic plasticity changes. However, muscle-specific data for the adductor magnus are still lacking. Should such modulation be confirmed, it could potentially contribute to the restoration of local inhibitory circuits within the spinal cord, thereby reestablishing the excitatory-inhibitory balance (37). Preliminary studies suggest a potential link between fascial elastic modulus and the excitability of motor neuron circuits (38, 39). Neuroimaging evidence indicates that acupuncture can enhance motor cortex excitability and improve sensorimotor integration processes (40, 41). Together, these mechanisms contribute to the repair of impaired motor pathways, facilitating motor function reorganization and the establishment of adaptive movement patterns.

### 3.3 Improvement in biomechanical compensation

Acupuncture intervention targeting the adductor magnus can restore the mechanical continuity of the DFL, thereby breaking the abnormal compensatory movement patterns resulting from post-stroke spasticity and facilitating the reestablishment of normal lower limb kinematics. When acupuncture alleviates spasticity of the adductor magnus, reduces abnormal tension in the tibialis posterior muscle, and releases inhibition of the peroneal muscle group, the ankle joint moments regain balance. Concretely manifested in three-dimensional gait analysis as a significant reduction in the foot inversion angle during the mid-stance phase, with the subtalar joint tending toward a functional neutral position. These changes not only correct compensatory postures such as femoral internal rotation and tibial posterior inclination but also significantly reduce abnormal impact forces on the lateral border of the foot during initial contact. Consequently, abnormal peak pressure in plantar pressure distribution is diminished, leading to improved walking stability and energy efficiency. During the swing phase of the gait cycle, enhanced function of the tibialis anterior muscle, coupled with appropriate activation of the peroneal muscles, effectively reduces toe drag and improves foot clearance. In the late stance phase, improved efficiency of push-off from the ankle joint facilitates active forward propulsion of the body, thereby increasing step frequency and stride length. More importantly, the restoration of overall tension within the DFL not only influences the ankle and foot but also helps reestablish synergistic coordination throughout the entire body’s kinetic chain. These holistic improvements can be objectively evaluated through enhanced trunk stability indices, optimized inter-joint energy transfer efficiency in the lower limbs, normalized plantar pressure distribution parameters, and elevated scores on standardized assessments such as the Berg Balance Scale and Dynamic Gait Index, thereby comprehensively promoting the restoration of motor function at both structural and functional levels following stroke.

## 4 Discussion

This study systematically proposes an integrative theoretical framework suggesting that “acupuncture applied to the adductor magnus modulates the DFL to improve equinovarus foot.” The potential mechanisms of action are elaborated from three perspectives: mechanical restructuring, neuroplasticity modulation, and biomechanical compensation. The innovative aspect of this framework lies in its integration of traditional acupuncture therapy with modern fascial chain theory, providing a novel holistic perspective for understanding acupuncture in treating post-stroke equinovarus foot. From a theoretical standpoint, this research extends beyond the limitations of conventional rehabilitation, which often focuses on isolated muscles or local joints, by emphasizing the central role of the fascial network in mechanical transmission and functional regulation. As a key pathway connecting core stability and distal function, the tension balance of the DFL is crucial for coordinating lower limb movement patterns. Critically, the anti-spastic effect of acupuncture, a foundation for this framework, is supported by clinical evidence. For instance, a multicenter randomized Controlled Trial demonstrated that aligned acupuncture at muscle regions significantly improved upper limb spasticity after stroke, with a total effective rate of 93.4% (42). Furthermore, a network meta-analysis of randomized trials specifically confirmed that acupoint-stimulating therapies, such as non-invasive electroacupuncture and warm needling, are effective in mitigating post-stroke spasticity in older survivors (43). This evidence underpins the proposed mechanism that reducing spasticity in key muscles like the adductor magnus can facilitate the restoration of mechanical balance along the DFL. However, the proposed theoretical framework has certain limitations. The DFL, as an anatomical concept, presents challenges for direct *in vivo* measurement and quantification of its mechanical transmission properties. Most current evidence is derived from anatomical observations and clinical inferences.

Based on this discussion, we propose the following future research directions: First, quantitative assessment of changes in DFL tension before and after acupuncture using techniques such as ultrasound imaging should be conducted. Second, the effects of acupuncture on lower limb muscle synergy patterns and gait parameters should be objectively evaluated through electromyography and kinematic analysis. Finally, multi-center, large-sample randomized controlled trials are warranted to verify the precise clinical efficacy of this treatment approach and to establish evidence-based therapeutic protocols.

## 5 Conclusion and prospects

In conclusion, despite its limitations, the theoretical framework proposed in this article offers a novel perspective for the rehabilitation of post-stroke equinovarus foot. While this integrative model highlights the potential of systematic fascial regulation, it remains hypothetical and requires rigorous validation. Future studies should focus on (1) quantifying DFL tension dynamics using shear-wave elastography, and (2) conducting comparative trials to evaluate the efficacy of adductor magnus acupuncture against conventional acupoint protocols. Through interdisciplinary collaboration and advanced biomechanical assessments, this approach may evolve into an evidence-based strategy to improve gait and functional outcomes in post-stroke rehabilitation.
